# Postoperative Ileus After Rectal Cancer Surgery: Assessing Incidence, Severity, and Impact Across Open, Laparoscopic, and Robotic Approaches

**DOI:** 10.3390/jcm15062295

**Published:** 2026-03-17

**Authors:** Michael Goldenshluger, Ofir Gruper, Yasmin Anderson, Tal Caller, Ephraim Katz, Alexander Lebedeyev, Ilan Kent, Edward Ram, Dean Lutrin, Lior Segev

**Affiliations:** 1 Faculty of Medical and Health Sciences, School of Medicine, Tel-Aviv University, Tel Aviv 69978, Israellior.segev@sheba.health.gov.il (L.S.); 2 Department of General Surgery, Chaim Sheba Medical Center, Ramat Gan 52621, Israel; 3 Neufled and Tamman Cardiovascular Research Institutes, Faculty of Medical and Health Sciences, Tel Aviv University, Tel Aviv 69978, Israel; 4 Lev Leviev Cardiovascular and Thoracic Center, Sheba Medical Center, Tel Hashomer, Ramat Gan 52621, Israel

**Keywords:** postoperative ileus, rectal cancer, rectal resection, minimally invasive surgery, laparoscopy, robotic surgery, open surgery, colorectal surgery, postoperative complications, risk factors

## Abstract

**Background/Objectives:** Postoperative paralytic ileus (POI) is a common complication after rectal resections. Although it is often argued that laparoscopic or robotic surgery reduces ileus compared to open surgery, research indicates that the incidence rates remain considerably high after minimally invasive surgery (MIS), and it is unclear whether laparoscopy or robotic surgery confers lower ileus rates. Furthermore, the literature lacks consistency in defining ileus and does not adequately address the severity levels of this complication. This study aims to compare the incidence and severity of ileus after open, laparoscopic, and robotic oncologic rectal resections, using definitions established in the current literature. **Methods:** This is a retrospective cohort study including patients who underwent rectal resection in a single tertiary academic-affiliated hospital between the years 2014 and 2019. The study compared patients who underwent laparoscopic and robotic surgeries to those who underwent open surgery. **Results:** The study involved 337 patients who underwent oncologic rectal resection. Among them, 188 underwent laparoscopic, 59 robotic and 90 underwent open surgery. The overall incidence of postoperative paralytic ileus (POI) was 19.6%, with a significant difference observed between surgical approaches: 15.4% in the MIS group compared to 31.1% in the open surgery group (*p* < 0.001). Additionally, a lower ileus incidence was noted between the robotic (8.5%) and laparoscopic (17.6%) subgroups, but it did not reach statistical significance (*p* = 0.092). The severity of ileus did not differ significantly between laparoscopic, robotic and open surgery. Ileus risk factors that were found included advanced age, male gender, high ASA score, preoperative anemia, intraoperative bowel injury, and postoperative opioid use. **Conclusions:** MIS for rectal cancer is linked to a significantly lower rate of POI compared to open surgery. However, when ileus does occur, its severity is comparable across all techniques.

## 1. Introduction

Post-operative ileus (POI) is a non-mechanical bowel disturbance caused by impaired intestinal motility, resulting from inflammation, neural reflexes, and neuro-humoral peptides [1,2]. Typically, ileus resolves within one to three days with supportive treatment, though it may persist for weeks in some cases, occasionally requiring full parenteral nutrition [3]. POI has a significant impact on the postoperative course, increasing the risk of complications, causing substantial patient discomfort, hindering mobilization, and prolonging hospitalization, leading to increased costs. The onset and progression of POI are difficult to predict. POI is defined as the period following surgery during which gas or stool is not passed, and oral diet tolerance is not achieved [4]. When these events occur early in postoperative recovery, they are considered a normal physiological response of the gastrointestinal tract to surgery. ‘Postoperative ileus’ (POI) refers to persistent ileus extending beyond the ‘obligatory POI’ phase and is independently associated with more severe symptoms. Although POI is commonly observed, there is no international consensus on its exact clinical definition [5]. The reported incidence of ileus varies widely in the literature. Up to 25% of patients experience some degree of ileus after elective abdominal surgeries, while POI occurs in 5–30% of patients following colorectal surgeries. Recent studies indicate that even with the advent of minimally invasive techniques, the incidence of ileus has not decreased after major oncological abdominal and pelvic surgeries, and in some cases, it has increased, particularly after colon and rectal resections and other cancer surgeries involving abdominal organs [6,7]. Although minimally invasive surgery is generally considered a protective factor against ileus in colorectal procedures, randomized controlled trials have shown only a modest effect of laparoscopic surgery on its incidence. Studies using the standard definition of ileus in the literature report significant rates, as high as 21%, in patients after laparoscopic surgeries [2]. The role of robotic surgery in preventing POI remains uncertain when compared to laparoscopy [8]. Moreover, the lack of a standardized clinical definition of ileus in previous studies hinders accurate assessment and evaluation of prevention and treatment strategies. This study aims to assess the incidence and severity of postoperative ileus following oncologic rectal resection surgeries. A secondary objective is to compare the occurrence and severity of ileus among open, laparoscopic, and robotic approaches. Based on existing literature, it is expected that open surgery will have the highest incidence and severity of ileus, followed by laparoscopic surgery, with robotic surgery showing the lowest rates. Additionally, the study seeks to identify the risk factors that are associated with postoperative ileus. Using data from this study, future research can focus on evaluating the effectiveness of treatments and preventive strategies for POI.

## 2. Methods

### 2.1. Patient Population

The study includes all patients who underwent rectal resection for cancer at a single tertiary referral center between January 2014 and December 2019. The exclusion criteria consisted of individuals under the age of 18, patients with metastatic disease, and those who underwent abdominoperineal resection (APR) or total proctocolectomy (TPC).

### 2.2. Variables

The variables in this study include both dependent and independent factors, as well as potential confounders.

The primary endpoint was postoperative ileus (POI). POI was defined as the occurrence of at least two gastrointestinal symptoms that started on postoperative day four or later, including nausea or vomiting, inability to tolerate oral intake for more than 24 h, absence of flatus or bowel movement for over 24 h, abdominal distension, or radiologic findings consistent with ileus.

This definition, initially described in the systematic review and global survey by Vather et al. [4], demonstrated ≥75% concordance among international experts and has since been widely adopted to standardize reporting. Accordingly, waiting until postoperative day 4 allows for a differentiation between the expected physiological postoperative gastrointestinal recovery and the clinically significant prolonged ileus. POI is classified as primary when no underlying cause, such as peritonitis or intra-abdominal bleeding, is identified, or secondary when it arises due to a complication like an anastomotic leak.

Ileus severity was categorized using the classification proposed by Venara et al. [9], which stratifies postoperative ileus according to clinical consequences, ranging from grade A (prolonged hospitalization only) to grade E (death). Grade B involves the need for symptomatic treatment or diagnostic procedures, including laxatives, prokinetic drugs, anti-spasmodic medications, and anti-nausea drugs. Grade C includes the requirement for nasogastric tube insertion or readmission after discharge, particularly if the tube is removed and reinserted. Grade D refers to severe complications, with D1 indicating renal dysfunction and D2 representing ICU admission or reoperation. The independent variables include the type of surgery, with a comparison between laparoscopic or robotic surgeries and open surgery.

Data on opioid use were collected from the medical team’s follow-up reports, summarizing the doses administered to each patient and standardized to the morphine equivalents. The opioid dose was treated as a continuous variable, allowing assessment of the dose–response relationship with the risk of postoperative ileus. Fluid administration volumes during the first four days post-surgery (up to post-operative day 4) were also documented in the medical records. Intraoperative bowel injury was defined based on the surgeon’s report, including any serosal injury, abrasion, partial serosal disruption, deeper bowel wall penetration, or perforation.

### 2.3. Surgical Technique

The type of surgery, either a low anterior resection of the rectum (LAR) or an anterior resection of the rectum (AR), was categorized based on the surgeon’s operative report. LAR was defined as a proctectomy with a total mesorectal excision (TME) down to the pelvic floor, while AR only involved a partial TME. Tumor height was measured in centimeters using rigid rectoscopy, with a classification as follows: a low rectal tumor (within 5 cm of the anal verge), a middle rectal tumor (5–10 cm), and an upper rectal tumor (10–20 cm).

### 2.4. Statistical Methods

Continuous variables were first assessed for distribution using the Kolmogorov–Smirnov test. Because most variables were not normally distributed, comparisons between groups were performed using the Mann–Whitney U test. Categorical variables were compared using the χ^2^ test. Continuous variables were presented as means with standard deviations, while dichotomous and categorical variables were presented as absolute numbers and percentages within each group. Ordinal and binary regression analyses were conducted to identify predictors of postoperative ileus and to assess its severity, both with and without adjustments for risk factors. Propensity scores were calculated for each patient, accounting for characteristics such as age at surgery, gender, BMI, smoking history, minimally invasive surgery status, and preoperative measures like albumin, hemoglobin, clinical staging, and cancer markers (CEA, CA19-9). The patients were matched based on these characteristics, which resulted in a matched cohort of 63 patients who underwent open surgery and 126 patients who underwent minimally invasive surgery. All of the statistical analyses were two-sided, with a significance level set at *p* < 0.05. The statistical analyses were performed using SPSS version 25.0 (SPSS Inc., Chicago, IL, USA), while the propensity score calculation and matching were performed using STATA BE V17 (StataCorp, College Station, TX, USA).

### 2.5. Ethics

This study was approved by the Helsinki Committee of Sheba Medical Center under the proposal number SMC-9618-22.

## 3. Results

### 3.1. Patient Characteristics

From January 2014 to December 2019, a total of 443 patients underwent anterior resection or low anterior resection at a single tertiary institution for rectal carcinoma treatment. After excluding 106 patients due to metastatic disease or additional colectomy or abdominoperineal resection, the final cohort comprised 337 rectal cancer patients. Among them, 188 underwent laparoscopic surgery, 59 had robotic surgery, and 90 underwent open surgery.

The cohort’s baseline characteristics are summarized in Table 1. The mean age of patients in the cohort was 63.6 years, with 56.4% (n = 190) being men. The tumor location varied, with 62.3% in the upper rectum, 31.5% in the middle rectum, and 6.2% in the lower rectum.

### 3.2. Incidence and Risk Factors for POI

The overall incidence of POI was 19.6% (n = 66). Open surgery had a higher POI rate of 31.1% (n = 28) compared to the minimally invasive group at 15.4% (n = 38). The laparoscopic and robotic surgeries were associated with a 57% lower risk of ileus (OR 0.43, 95% CI 0.24–0.76, *p* = 0.004) (Table 2). While robotic surgery showed a lower ileus rate compared to laparoscopic surgery (8.5% vs. 17.6%, *p* = 0.092), the difference was not statistically significant. The mean preoperative hemoglobin (Hb) level was significantly lower among patients who developed POI compared to those who did not. Specifically, the mean Hb was 11.95 g/dL in the POI group versus 12.78 g/dL in the non-POI group, corresponding to a mean difference of 0.83 g/dL (*p* < 0.05).

Univariate analysis identified significant risk factors for POI, including older age (OR 1.68, *p* = 0.03), male gender (OR 1.92, *p* = 0.049), higher ASA score (OR 1.74, *p* = 0.036), and low preoperative hemoglobin (OR 2.4, *p* = 0.052). Surgical factors like intraoperative bowel injury (OR 5.86, *p* = 0.031) and high-dose opioid use (OR 1.58, *p* < 0.001) were also associated with increased POI risk.

We compared the differences in the severity of ileus between the definition found in the literature (occurrence of two or more symptoms starting from postoperative day 4: vomiting; inability to tolerate oral nutrition for more than 24 h; absence of gas passage for more than 24 h; abdominal distension; and a characteristic radiographic image) and the clinical diagnosis. Among the 337 patients in the study, 66 patients (19.6%) met the literature-based criteria for ileus diagnosis, although only 33 (9.8%) were clinically diagnosed with POI by the medical team. Of the patients who met the literature-based definition for POI, 31 (47%) were also identified by the medical team, while 35 patients (53%) were not clinically diagnosed despite meeting the literature-defined criteria. Only 2 patients (0.7%) who were clinically diagnosed with ileus did not meet the literature-based criteria.

### 3.3. Complications and Surgical Outcomes

The binary logistic regression that was adjusted for age, gender, BMI, and ASA score confirmed that open surgery was an independent risk factor for ileus (OR 0.44, 95% CI 0.24–0.8, *p* = 0.007). A per-protocol analysis comparing planned open surgeries to converted minimally invasive surgeries showed no significant difference in ileus risk (OR 0.99, *p* = 0.985). Secondary analysis using propensity score matching (63 open surgeries vs. 126 minimally invasive surgeries) confirmed lower ileus rates in the minimally invasive group (17.5% vs. 35%, OR 0.39, *p* = 0.008). We specifically examined whether the conversion from minimally invasive to open surgery was associated with an increased risk of POI using a per-protocol analysis comparing planned open procedures with cases that were initiated minimally invasively and converted to open. In this analysis, conversion was not associated with an increased risk of POI (OR 0.99, 95% CI 0.47–2.08, *p* = 0.985), suggesting that the surgical approach that was ultimately performed is the primary determinant of POI risk.

### 3.4. Ileus Severity and Management

Among the 66 patients who developed ileus, severity classifications included 32% as grade A, 15% as grade B, and 47% as grade C. Severe cases were less frequent, with 4.5% in grade D and 1.5% in grade E. Ordinal logistic regression showed no significant difference in ileus severity between open and minimally invasive surgery (OR 1.19, *p* = 0.182).

### 3.5. Additional Clinical Benefits

**Figure 1:**
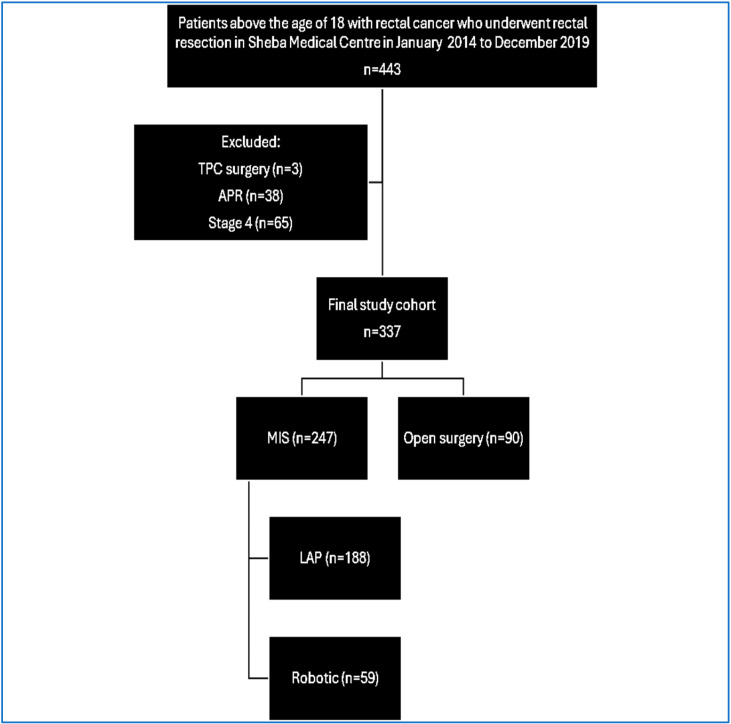
Consort chart. Abbreviations: TPC: total proctocolectomy, APR: abdominoperineal resection, MIS: minimally invasive surgery, and LAP: laparoscopy.

**Figure 2:**
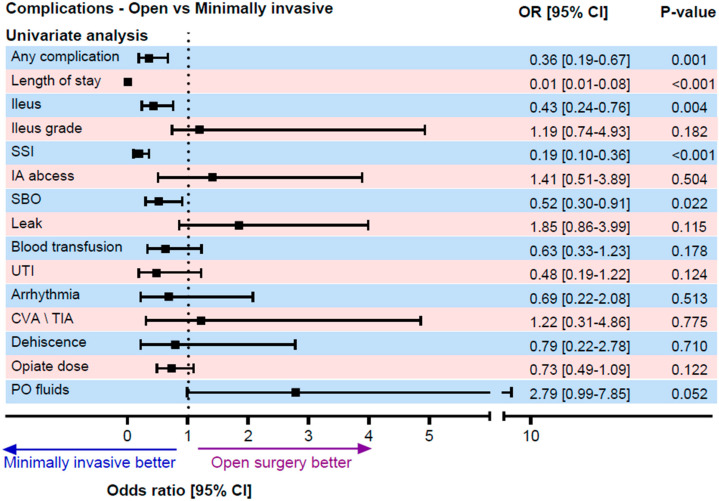
Postoperative complications (minimally invasive vs. open surgery), univariate analysis. The odds ratios and *p* values were derived using logistic regression for categorical outcomes and linear regression for continuous outcomes. Abbreviations: SSI: surgical site infection, IA abscess: intra-abdominal abscess, UTI: urinary tract infection, CVA/TIA: cerebrovascular accident/transient ischemic attack, and PO: postoperative.

**Figure 3:**
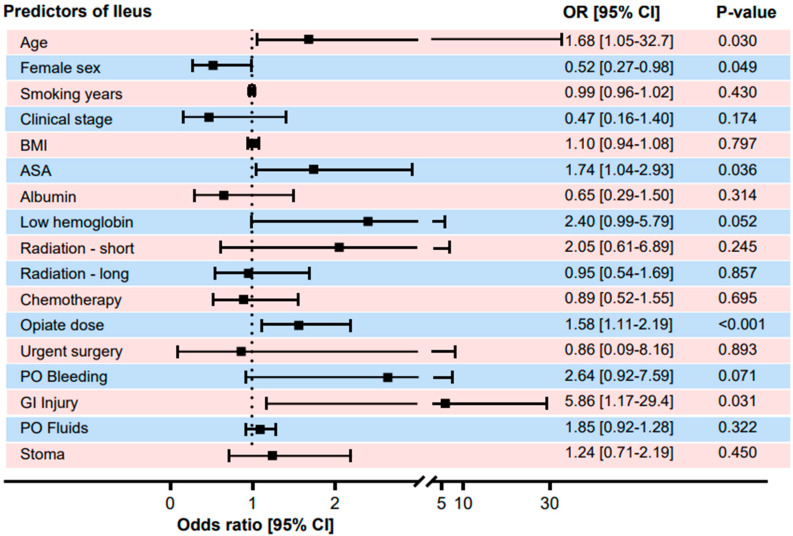
Predictive factors for postoperative ileus. The *p* values and odds ratios were calculated using binary logistic regression for categorical variables and linear regression for continuous variables. Abbreviations: BMI: body mass index, ASA: American Society of Anesthesiologists, PO: postoperative, and GI: gastrointestinal.

**Figure 4:**
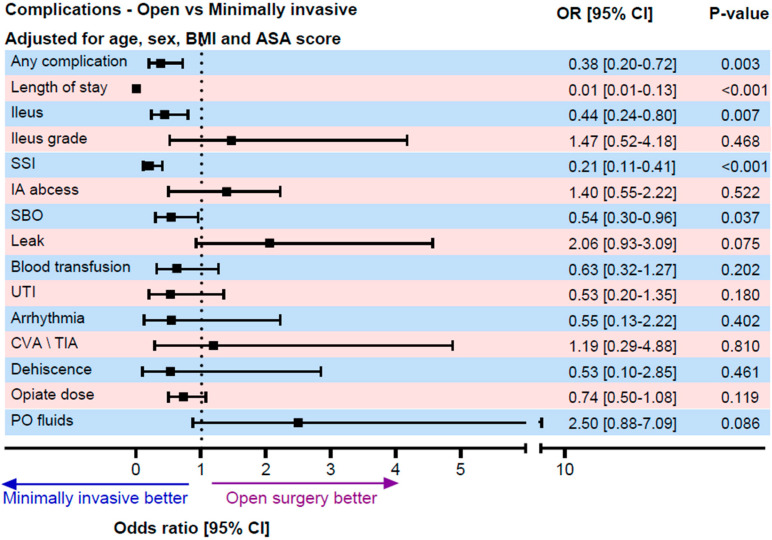
Postoperative complications (minimally invasive vs. open surgery), multivariate analysis adjusted for age, gender, BMI, and ASA Score. The *p* value and odds ratio were calculated using binary/ordinal logistic regression for categorical variables and linear regression for continuous variables. The model was adjusted for age, gender, BMI, and ASA score. Abbreviations: SSI: surgical site infection, IA abscess: intra-abdominal abscess, UTI: urinary tract infection, CVA/TIA: cerebrovascular accident/transient ischemic attack, and PO: postoperative.

Minimally invasive surgery demonstrated several additional benefits, including a 62% reduction in overall complications, halving the hospital stay duration, an 81% reduction in surgical site infections, and a 46% reduction in small bowel obstruction. After adjusting for confounders, the only statistically significant benefit that remained was the reduced hospital stay duration. No significant correlation was found between the surgery type and the complications, such as postoperative fluid collections, intra-abdominal abscesses, blood transfusion requirements, and opioid use (Figure 1, Figure 2, Figure 3 and Figure 4).

## 4. Discussion

The key findings of this study indicate a significantly lower incidence of ileus in minimally invasive rectal resections, with a trend suggesting a potential advantage of using robotic surgery over laparoscopy. Notably, anemia was identified as a major risk factor for ileus, a factor not previously reported in the literature.

This study confirms, through multivariate analysis and propensity score matching, that minimally invasive techniques act as a protective factor against POI. While non-randomized studies have linked robotic surgery to a significantly lower risk of ileus compared to the laparoscopic approach, a meta-analysis of randomized controlled trials did not reveal a significant difference between the two methods [10,11]. Our findings indicate a trend toward reduced ileus in the robotic surgery group. Notably, patients in the robotic group underwent more low resections and had higher rates of preoperative radiation—both factors that increase surgical complexity—further reinforcing the protective effect of robotic surgery against POI.

Our analysis of other risk factors for POI confirmed that age is a significant predictor, aligning with the existing literature [7]. Our study demonstrated that among middle-aged patients, the risk of developing ileus increased by 70% with each additional decade of life. Other established risk factors, including male sex, high American Society of Anesthesiologists (ASA) scores, and elevated postoperative opioid use, were also corroborated [12,13].

Notably, this study identified low preoperative hemoglobin levels as a previously unrecognized risk factor for POI. However, preoperative anemia is well-documented as a predictor of postoperative complications, increased 30-day morbidity, and prolonged hospital stay. Several pathophysiological mechanisms may underlie the association between preoperative anemia and ileus. Chronic inflammation, particularly in cases of iron deficiency anemia, may amplify the postoperative inflammatory response, while tissue hypoxia can impair wound healing and bowel motility. Additionally, perioperative blood transfusions, which are often required in anemic patients, have been independently associated with an increased risk of ileus.

Although prior studies have identified diverting stomas, particularly ileostomies, as a risk factor for POI^11^, we did not observe this association in our cohort. This may suggest that other factors influencing postoperative recovery in our population have had a greater impact than the presence of a stoma itself.

In this study, we report a relatively high POI incidence of almost 20% in a large cohort of oncologic proctectomy patients. This rate is higher than that reported for colorectal surgeries (9.4–14.2%) but lower than what is observed in randomized controlled trials that focus solely on rectal resection (30.9%) [13].

A potential explanation for the higher incidence of POI in this study is the definition applied. When classified based on the medical teams’ assessment, the incidence was 9.8%, whereas it increased to 19.6% when defined strictly according to the literature criteria. Notably, 53% of patients meeting the formal literature-based definition of ileus were not recognized as having the condition by the medical team. The study findings indicate that most of these additional cases identified by the literature definition were of mild severity. These included cases where ileus resulted solely in prolonged hospitalization (grade A) or required symptomatic management or a diagnostic evaluation (grade B). It appears that the medical team, when assessing these mild cases, attributed the symptoms to physiological POI—an expected postoperative occurrence that typically resolves without significant consequences. The predominance of mild cases among those diagnosed using the literature definition suggests that exclusive reliance on this criterion could lead to an overdiagnosis of clinically insignificant cases. These findings underscore the necessity for a more refined diagnostic approach to ileus, where clinical judgment plays a role in distinguishing mild cases from those with meaningful clinical implications.

Meta-analyses conducted by Vather et al. (2013) have highlighted inconsistencies in the terminology used across POI studies [4]. Specifically, the differentiation between “physiological” ileus and POI, which is more likely to have significant clinical consequences, is often unclear [4].

Another factor to consider is that during the period in which this study was conducted, an enhanced recovery after surgery (ERAS) protocol had not yet been fully implemented in our practice, which may have contributed to the higher frequency of postoperative ileus that was observed in this cohort.

This study is limited by its retrospective observational nature, which could introduce secondary biases due to confounding factors. Additionally, this is a single-center study, which reduces its external validity. In this context, there may have been a loss to follow-up for patients who received treatment at other hospitals due to complications. Moreover, the study relies on medical records that were written for clinical purposes, not research purposes, and thus some data were missing, potentially affecting the results. For example, there were no records on surgical time or blood loss during surgery, both of which are known risk factors for postoperative ileus. Furthermore, in the earlier years of the study (2014), there was no systematic documentation of the opioid doses given after surgery, meaning the true effect of opioid administration on ileus development may have been underestimated.

Regarding the analysis demonstrating a lower rate of POI in the robotic group compared with the open surgery group, this finding should be interpreted with caution, as the analysis may be underpowered. The sample size within the robotic subgroup may have been insufficient to detect a statistically significant difference.

Finally, our multivariable model may not have accounted for all potential risk factors and confounders, which is a common limitation in observational studies. In particular, operative complexity variables such as the extent of mesorectal excision were not included and may have influenced surgical difficulty and postoperative recovery.

Nonetheless, the study has several strengths. First, we employed multiple statistical methods for both primary and secondary analyses to validate the findings. Although it appeared that patients in the minimally invasive surgery (MIS) group were younger compared to those in the open surgery group, after adjusting and matching for confounders, we were able to demonstrate that MIS is associated with a lower incidence of ileus. Second, all participants included in the study underwent blinded adjudication. The chart review was manual, ensuring that data and diagnoses were accurate. Third, the fact that the study focused solely on rectal resection is a strength. Many studies examining the incidence of ileus combine rectal and colon surgeries, which could lower the reported incidence.

## 5. Conclusions

In conclusion, MIS for rectal cancer is associated with a significantly lower risk of postoperative ileus compared to open surgery, with a notable trend suggesting that robotic surgery may offer further advantages over laparoscopy. However, when ileus occurs, its severity remains comparable between the two techniques. It is important to note that this study identified preoperative anemia as a previously unrecognized risk factor for postoperative ileus, emphasizing the need for preoperative optimization to mitigate this risk.

Furthermore, the discrepancy between clinically diagnosed ileus and cases identified using literature-based criteria highlights the potential for overdiagnosis when relying solely on rigid definitions. Incorporating clinical judgment into the diagnostic process is essential to distinguish mild, self-resolving cases from those with meaningful clinical consequences.

## Figures and Tables

**Table 1:** Demographic Characteristics and Comorbidities.

	All Patients (n = 337)	Open Surgery (n = 90)	Laparoscopic Surgery (n = 188)	Robotic Surgery (n = 59)	*p* Value
Age	63.6 ± 12.0	66.2 ± 12.0	62.5 ± 12.1	62.2 ± 11	0.4
Female Sex	147 (43.6%)	42 (46.7%)	83 (44.1%)	22 (37.3%)	0.5
BMI	26.4 ± 4.5	27.3 ± 5.1	26.2 ± 4.2	26.1 ± 4.0	0.3
ASA Score					0.08
I	17 (5.2%)	3 (1.8%)	12 (6.4%)	2 (3.4%)	
II	115 (35.5%)	32 (28.8%)	45 (23.9%)	6 (10.2%)	
III	182 (56.2%)	45 (50%)	110 (58.5%)	27 (45.8%)	
IV	10 (3.1%)	6 (5.4%)	2 (1.1%)	2 (3.4%)	
Clinical Stage					0.07
Stage 1	87(25.8%)	23 (25.6%)	58 (30.9%)	6 (10.2%)	
Stage 2	48 (14.2%)	14 (15.6%)	24 (12.8%)	10 (16.9%)	
Stage 3	174 (51.6%)	43 (47.8%)	93 (49.5%)	38 (64.4%)	
TIA/CVA	11 (3.3%)	5 (5.6%)	4 (4.4%)	2 (1.1%)	0.3
Asthma/COPD	28 (8.3%)	8 (8.9%)			
IHD_CHF	42 (12.5%)	14 (15.6%)	22 (11.7%)	6 (10.2%)	0.6
DM	71 (21.1%)	23 (25.6%)	35 (38.9%)	13 (22%)	0.4
CRF	11 (3.3%)	6 (6.7%)	3 (3.3%)	2 (1.1%)	0.08
HTN	149 (44.2%)	46 (51.1%)	84 (44.5%)	19 (32.2%)	0.08
Smoking					0.3
Current	62 (18.4%)	15 (16.7%)	33 (17.6%)	14 (23.7%)	
Smoke Years	30.3 (25.9)	35.9 (21.4)	28.4 (28.4)	29 (24.8)	
History of Abdominal Surgery	134 (39.8%)	34 (37.8%)	72 (38.3%	28 (47.5%)	0.4
Pre-operative Radiation					0.02
Short Dose	13 (3.9%)	2 (2.2%)	4 (2.2%)	7 (11.9%)	
Long Dose	134 (39.8%)	37 (41.1%)	67 (35.6%)	30 (50.8%)	
Pre-operative chemotherapy	197 (58.5%)	50 (55.6%)	121 (64.4%)	26 (44.1%)	0.02
Type of Surgery LAR Dissection Below Peritoneal Reflection (LAR)	192 (57%)	53 (58.9%)	98 (52.1%)	41 (69.5%)	0.06

*p* values for categorical variables were calculated using the chi-square test, and results are presented as counts with corresponding percentages. For continuous data, *p* values were calculated using the non-parametric Mann–Whitney U test, and the data were presented as the mean (standard deviation). Abbreviations: BMI: body mass index, TIA/CVA: cerebrovascular accident/transient ischemic attack, COPD: chronic obstructive pulmonary disease, IHD_CHF: ischemic heart disease/congestive heart failure, DM: diabetes mellitus, CRF: chronic renal failure, HTN: hypertension, LAR: low anterior resection.

**Table 2:** Incidence of ileus in laparoscopic vs. robotic surgery. *p* value for categorical data was calculated using the χ^2^ test, and data were presented as raw numbers (percentages). Postoperative Ileus by Surgical Approach.

	Ileus	No Ileus	Total
Laparoscopic	33 (17.6%)	155 (82.4%)	188
Robotic	5 (8.5%)	54 (91.5%)	59
Total	38 (15.4%)	209 (84.6%)	247

*p* (chi-square) = 0.092.

## Data Availability

The data supporting the findings of this study are available from the corresponding author upon reasonable request. The data are not publicly available due to privacy and ethical restrictions.
